# Supplementary figures and images for: Quantification of left ventricular mass in multiple views of echocardiograms using model-agnostic meta learning in a few-shot setting (part 1 of 2)
Source: PeerJ Comput Sci. 2025 Sep 16;11:e3161. doi: 10.7717/peerj-cs.3161 (PMC12453733; doi:10.7717/peerj-cs.3161)

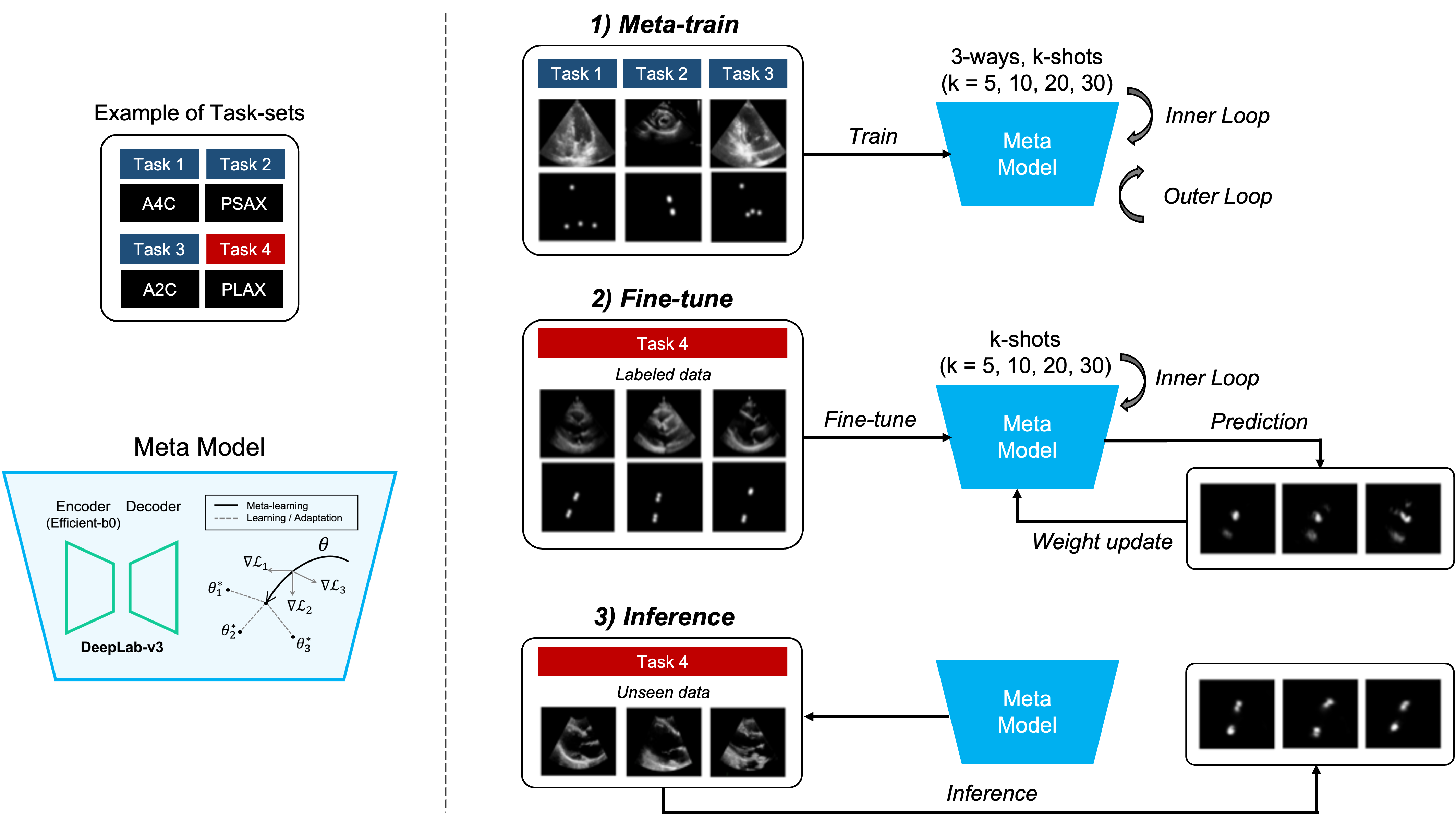

Supplement: Supplemental Information 1 [file peerj-cs-11-3161-s001.zip › code/Figure 1.png]

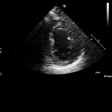

Supplement: Supplemental Information 2 [file peerj-cs-11-3161-s002.zip › PSAX/train_100/1614s1_38.png]

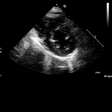

Supplement: Supplemental Information 2 [file peerj-cs-11-3161-s002.zip › PSAX/train_100/1477s1_26.png]

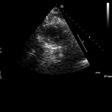

Supplement: Supplemental Information 2 [file peerj-cs-11-3161-s002.zip › PSAX/train_100/1641s1_29.png]

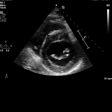

Supplement: Supplemental Information 2 [file peerj-cs-11-3161-s002.zip › PSAX/train_100/2038s1_65.png]

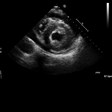

Supplement: Supplemental Information 2 [file peerj-cs-11-3161-s002.zip › PSAX/train_100/1754s1_11.png]

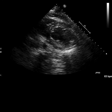

Supplement: Supplemental Information 2 [file peerj-cs-11-3161-s002.zip › PSAX/train_100/1082s1_10.png]

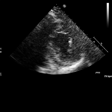

Supplement: Supplemental Information 2 [file peerj-cs-11-3161-s002.zip › PSAX/train_100/1614s1_39.png]

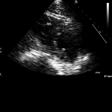

Supplement: Supplemental Information 2 [file peerj-cs-11-3161-s002.zip › PSAX/train_100/1490s2_26.png]

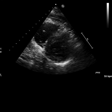

Supplement: Supplemental Information 2 [file peerj-cs-11-3161-s002.zip › PSAX/train_100/1393s1_57.png]

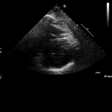

Supplement: Supplemental Information 2 [file peerj-cs-11-3161-s002.zip › PSAX/train_100/1873s1_68.png]

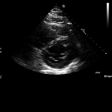

Supplement: Supplemental Information 2 [file peerj-cs-11-3161-s002.zip › PSAX/train_100/1851s3_55.png]

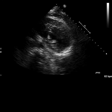

Supplement: Supplemental Information 2 [file peerj-cs-11-3161-s002.zip › PSAX/train_100/1082s1_12.png]

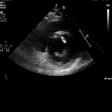

Supplement: Supplemental Information 2 [file peerj-cs-11-3161-s002.zip › PSAX/train_100/2038s1_66.png]

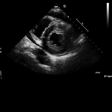

Supplement: Supplemental Information 2 [file peerj-cs-11-3161-s002.zip › PSAX/train_100/1754s1_12.png]

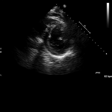

Supplement: Supplemental Information 2 [file peerj-cs-11-3161-s002.zip › PSAX/train_100/1082s1_13.png]

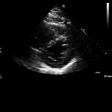

Supplement: Supplemental Information 2 [file peerj-cs-11-3161-s002.zip › PSAX/train_100/1851s3_54.png]

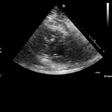

Supplement: Supplemental Information 2 [file peerj-cs-11-3161-s002.zip › PSAX/train_100/1089s1_38.png]

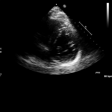

Supplement: Supplemental Information 2 [file peerj-cs-11-3161-s002.zip › PSAX/train_100/1071s1_28.png]

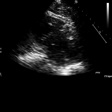

Supplement: Supplemental Information 2 [file peerj-cs-11-3161-s002.zip › PSAX/train_100/1490s2_27.png]

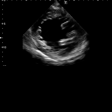

Supplement: Supplemental Information 2 [file peerj-cs-11-3161-s002.zip › PSAX/train_100/1508s1_21.png]

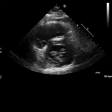

Supplement: Supplemental Information 2 [file peerj-cs-11-3161-s002.zip › PSAX/train_100/1873s1_51.png]

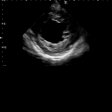

Supplement: Supplemental Information 2 [file peerj-cs-11-3161-s002.zip › PSAX/train_100/1508s1_20.png]

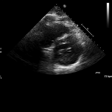

Supplement: Supplemental Information 2 [file peerj-cs-11-3161-s002.zip › PSAX/train_100/1873s1_50.png]

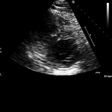

Supplement: Supplemental Information 2 [file peerj-cs-11-3161-s002.zip › PSAX/train_100/1490s2_36.png]

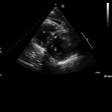

Supplement: Supplemental Information 2 [file peerj-cs-11-3161-s002.zip › PSAX/train_100/1393s1_45.png]

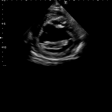

Supplement: Supplemental Information 2 [file peerj-cs-11-3161-s002.zip › PSAX/train_100/1508s1_22.png]

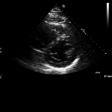

Supplement: Supplemental Information 2 [file peerj-cs-11-3161-s002.zip › PSAX/train_100/1851s3_53.png]

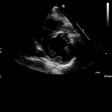

Supplement: Supplemental Information 2 [file peerj-cs-11-3161-s002.zip › PSAX/train_100/1159s1_50.png]

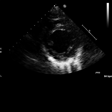

Supplement: Supplemental Information 2 [file peerj-cs-11-3161-s002.zip › PSAX/train_100/1205s1_37.png]

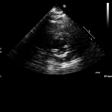

Supplement: Supplemental Information 2 [file peerj-cs-11-3161-s002.zip › PSAX/train_100/2013s1_20.png]

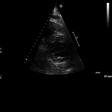

Supplement: Supplemental Information 2 [file peerj-cs-11-3161-s002.zip › PSAX/train_100/1935s1_37.png]

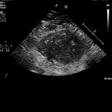

Supplement: Supplemental Information 2 [file peerj-cs-11-3161-s002.zip › PSAX/train_100/2038s1_61.png]

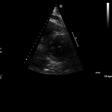

Supplement: Supplemental Information 2 [file peerj-cs-11-3161-s002.zip › PSAX/train_100/1935s1_36.png]

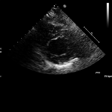

Supplement: Supplemental Information 2 [file peerj-cs-11-3161-s002.zip › PSAX/train_100/1205s1_36.png]

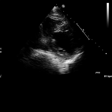

Supplement: Supplemental Information 2 [file peerj-cs-11-3161-s002.zip › PSAX/train_100/1159s1_51.png]

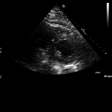

Supplement: Supplemental Information 2 [file peerj-cs-11-3161-s002.zip › PSAX/train_100/1851s3_52.png]

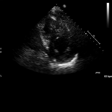

Supplement: Supplemental Information 2 [file peerj-cs-11-3161-s002.zip › PSAX/train_100/1666s1_44.png]

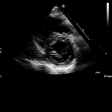

Supplement: Supplemental Information 2 [file peerj-cs-11-3161-s002.zip › PSAX/train_100/1159s1_22.png]

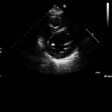

Supplement: Supplemental Information 2 [file peerj-cs-11-3161-s002.zip › PSAX/train_100/1205s1_51.png]

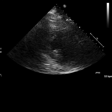

Supplement: Supplemental Information 2 [file peerj-cs-11-3161-s002.zip › PSAX/train_100/1422s1_68.png]

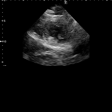

Supplement: Supplemental Information 2 [file peerj-cs-11-3161-s002.zip › PSAX/train_100/1422s1_40.png]

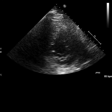

Supplement: Supplemental Information 2 [file peerj-cs-11-3161-s002.zip › PSAX/train_100/1422s1_69.png]

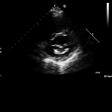

Supplement: Supplemental Information 2 [file peerj-cs-11-3161-s002.zip › PSAX/train_100/1205s1_50.png]

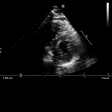

Supplement: Supplemental Information 2 [file peerj-cs-11-3161-s002.zip › PSAX/train_100/1984s1_40.png]

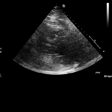

Supplement: Supplemental Information 2 [file peerj-cs-11-3161-s002.zip › PSAX/train_100/1089s1_58.png]

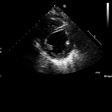

Supplement: Supplemental Information 2 [file peerj-cs-11-3161-s002.zip › PSAX/train_100/1205s1_52.png]

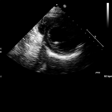

Supplement: Supplemental Information 2 [file peerj-cs-11-3161-s002.zip › PSAX/train_100/1477s1_8.png]

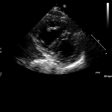

Supplement: Supplemental Information 2 [file peerj-cs-11-3161-s002.zip › PSAX/train_100/1310s1_52.png]

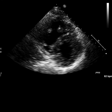

Supplement: Supplemental Information 2 [file peerj-cs-11-3161-s002.zip › PSAX/train_100/1310s1_53.png]

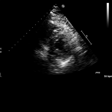

Supplement: Supplemental Information 2 [file peerj-cs-11-3161-s002.zip › PSAX/train_100/1984s1_46.png]

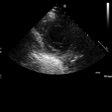

Supplement: Supplemental Information 2 [file peerj-cs-11-3161-s002.zip › PSAX/train_100/1111s1_25.png]

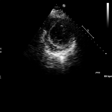

Supplement: Supplemental Information 2 [file peerj-cs-11-3161-s002.zip › PSAX/train_100/1040s1_50.png]

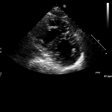

Supplement: Supplemental Information 2 [file peerj-cs-11-3161-s002.zip › PSAX/train_100/1310s1_51.png]

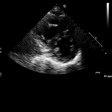

Supplement: Supplemental Information 2 [file peerj-cs-11-3161-s002.zip › PSAX/train_100/1310s1_50.png]

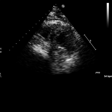

Supplement: Supplemental Information 2 [file peerj-cs-11-3161-s002.zip › PSAX/train_100/1984s1_45.png]

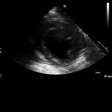

Supplement: Supplemental Information 2 [file peerj-cs-11-3161-s002.zip › PSAX/train_100/1111s1_26.png]

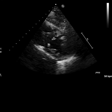

Supplement: Supplemental Information 2 [file peerj-cs-11-3161-s002.zip › PSAX/train_100/1393s1_33.png]

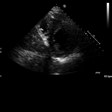

Supplement: Supplemental Information 2 [file peerj-cs-11-3161-s002.zip › PSAX/train_100/1666s1_40.png]

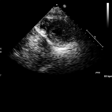

Supplement: Supplemental Information 2 [file peerj-cs-11-3161-s002.zip › PSAX/train_100/1477s1_7.png]

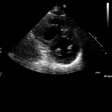

Supplement: Supplemental Information 2 [file peerj-cs-11-3161-s002.zip › PSAX/train_100/1310s1_49.png]

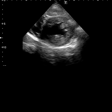

Supplement: Supplemental Information 2 [file peerj-cs-11-3161-s002.zip › PSAX/train_100/1099s1_32.png]

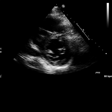

Supplement: Supplemental Information 2 [file peerj-cs-11-3161-s002.zip › PSAX/train_100/1159s1_16.png]

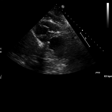

Supplement: Supplemental Information 2 [file peerj-cs-11-3161-s002.zip › PSAX/train_100/1991s1_34.png]

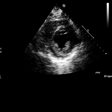

Supplement: Supplemental Information 2 [file peerj-cs-11-3161-s002.zip › PSAX/train_100/1040s1_49.png]

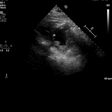

Supplement: Supplemental Information 2 [file peerj-cs-11-3161-s002.zip › PSAX/train_100/2200s1_36.png]

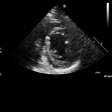

Supplement: Supplemental Information 2 [file peerj-cs-11-3161-s002.zip › PSAX/train_100/1614s1_40.png]

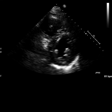

Supplement: Supplemental Information 2 [file peerj-cs-11-3161-s002.zip › PSAX/train_100/1666s2_26.png]

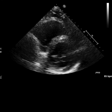

Supplement: Supplemental Information 2 [file peerj-cs-11-3161-s002.zip › PSAX/train_100/1991s1_33.png]

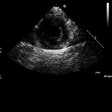

Supplement: Supplemental Information 2 [file peerj-cs-11-3161-s002.zip › PSAX/train_100/1618s1_32.png]

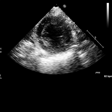

Supplement: Supplemental Information 2 [file peerj-cs-11-3161-s002.zip › PSAX/train_100/1618s1_33.png]

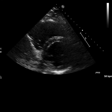

Supplement: Supplemental Information 2 [file peerj-cs-11-3161-s002.zip › PSAX/train_100/1991s1_32.png]

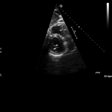

Supplement: Supplemental Information 2 [file peerj-cs-11-3161-s002.zip › PSAX/train_100/1393s1_39.png]

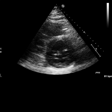

Supplement: Supplemental Information 2 [file peerj-cs-11-3161-s002.zip › PSAX/train_100/1089s1_57.png]

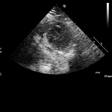

Supplement: Supplemental Information 2 [file peerj-cs-11-3161-s002.zip › PSAX/train_100/1614s1_41.png]

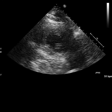

Supplement: Supplemental Information 2 [file peerj-cs-11-3161-s002.zip › PSAX/train_100/1422s1_70.png]

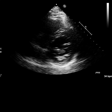

Supplement: Supplemental Information 2 [file peerj-cs-11-3161-s002.zip › PSAX/train_100/1071s1_44.png]

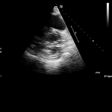

Supplement: Supplemental Information 2 [file peerj-cs-11-3161-s002.zip › PSAX/train_100/1621s2_76.png]

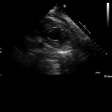

Supplement: Supplemental Information 2 [file peerj-cs-11-3161-s002.zip › PSAX/train_100/1082s1_18.png]

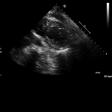

Supplement: Supplemental Information 2 [file peerj-cs-11-3161-s002.zip › PSAX/train_100/1082s1_30.png]

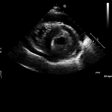

Supplement: Supplemental Information 2 [file peerj-cs-11-3161-s002.zip › PSAX/train_100/1754s1_25.png]

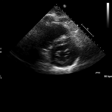

Supplement: Supplemental Information 2 [file peerj-cs-11-3161-s002.zip › PSAX/train_100/1873s1_49.png]

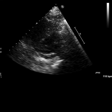

Supplement: Supplemental Information 2 [file peerj-cs-11-3161-s002.zip › PSAX/train_100/1759s1_40.png]

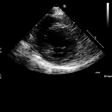

Supplement: Supplemental Information 2 [file peerj-cs-11-3161-s002.zip › PSAX/train_100/1618s1_54.png]

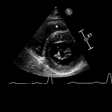

Supplement: Supplemental Information 2 [file peerj-cs-11-3161-s002.zip › PSAX/train_100/2038s1_47.png]

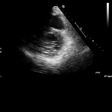

Supplement: Supplemental Information 2 [file peerj-cs-11-3161-s002.zip › PSAX/train_100/1621s2_74.png]

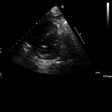

Supplement: Supplemental Information 2 [file peerj-cs-11-3161-s002.zip › PSAX/train_100/1759s1_41.png]

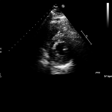

Supplement: Supplemental Information 2 [file peerj-cs-11-3161-s002.zip › PSAX/train_100/1984s1_39.png]

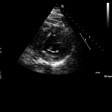

Supplement: Supplemental Information 2 [file peerj-cs-11-3161-s002.zip › PSAX/train_100/1641s1_32.png]

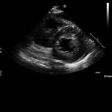

Supplement: Supplemental Information 2 [file peerj-cs-11-3161-s002.zip › PSAX/train_100/1754s1_23.png]

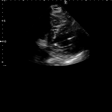

Supplement: Supplemental Information 2 [file peerj-cs-11-3161-s002.zip › PSAX/train_100/1422s1_38.png]

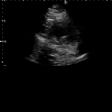

Supplement: Supplemental Information 2 [file peerj-cs-11-3161-s002.zip › PSAX/train_100/1422s1_39.png]

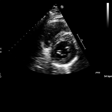

Supplement: Supplemental Information 2 [file peerj-cs-11-3161-s002.zip › PSAX/train_100/1984s1_38.png]

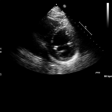

Supplement: Supplemental Information 2 [file peerj-cs-11-3161-s002.zip › PSAX/train_100/1071s1_26.png]

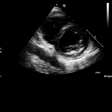

Supplement: Supplemental Information 2 [file peerj-cs-11-3161-s002.zip › PSAX/train_100/1477s1_16.png]

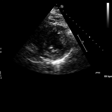

Supplement: Supplemental Information 2 [file peerj-cs-11-3161-s002.zip › PSAX/train_100/1641s1_31.png]

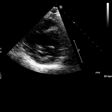

Supplement: Supplemental Information 2 [file peerj-cs-11-3161-s002.zip › PSAX/train_100/1618s1_47.png]

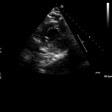

Supplement: Supplemental Information 2 [file peerj-cs-11-3161-s002.zip › PSAX/train_100/1641s1_30.png]

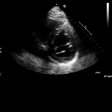

Supplement: Supplemental Information 2 [file peerj-cs-11-3161-s002.zip › PSAX/train_100/1071s1_27.png]

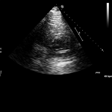

Supplement: Supplemental Information 2 [file peerj-cs-11-3161-s002.zip › PSAX/test/2453s1_34.png]
