# Supplementary figures and images for: Quantification of left ventricular mass in multiple views of echocardiograms using model-agnostic meta learning in a few-shot setting (part 2 of 2)
Source: PeerJ Comput Sci. 2025 Sep 16;11:e3161. doi: 10.7717/peerj-cs.3161 (PMC12453733; doi:10.7717/peerj-cs.3161)

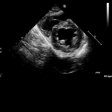

Supplement: Supplemental Information 2 [file peerj-cs-11-3161-s002.zip › PSAX/test/2398s1_18.png]

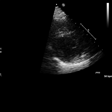

Supplement: Supplemental Information 2 [file peerj-cs-11-3161-s002.zip › PSAX/test/2479s2_25.png]

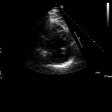

Supplement: Supplemental Information 2 [file peerj-cs-11-3161-s002.zip › PSAX/test/2241s1_27.png]

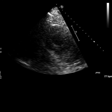

Supplement: Supplemental Information 2 [file peerj-cs-11-3161-s002.zip › PSAX/test/2241s1_33.png]

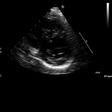

Supplement: Supplemental Information 2 [file peerj-cs-11-3161-s002.zip › PSAX/test/3283s1_53.png]

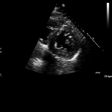

Supplement: Supplemental Information 2 [file peerj-cs-11-3161-s002.zip › PSAX/test/3492s3_28.png]

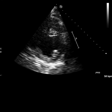

Supplement: Supplemental Information 2 [file peerj-cs-11-3161-s002.zip › PSAX/test/3153s1_48.png]

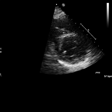

Supplement: Supplemental Information 2 [file peerj-cs-11-3161-s002.zip › PSAX/test/2479s2_26.png]

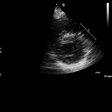

Supplement: Supplemental Information 2 [file peerj-cs-11-3161-s002.zip › PSAX/test/2479s2_27.png]

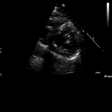

Supplement: Supplemental Information 2 [file peerj-cs-11-3161-s002.zip › PSAX/test/3492s3_29.png]

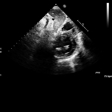

Supplement: Supplemental Information 2 [file peerj-cs-11-3161-s002.zip › PSAX/test/3223s1_31.png]

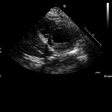

Supplement: Supplemental Information 2 [file peerj-cs-11-3161-s002.zip › PSAX/test/3283s1_56.png]

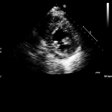

Supplement: Supplemental Information 2 [file peerj-cs-11-3161-s002.zip › PSAX/test/3325s1_63.png]

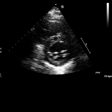

Supplement: Supplemental Information 2 [file peerj-cs-11-3161-s002.zip › PSAX/test/2281s1_18.png]

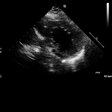

Supplement: Supplemental Information 2 [file peerj-cs-11-3161-s002.zip › PSAX/test/3339s2_37.png]

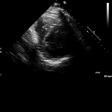

Supplement: Supplemental Information 2 [file peerj-cs-11-3161-s002.zip › PSAX/test/3375s1_50.png]

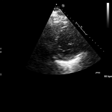

Supplement: Supplemental Information 2 [file peerj-cs-11-3161-s002.zip › PSAX/test/3305s1_21.png]

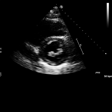

Supplement: Supplemental Information 2 [file peerj-cs-11-3161-s002.zip › PSAX/test/3190s1_32.png]

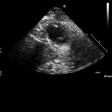

Supplement: Supplemental Information 2 [file peerj-cs-11-3161-s002.zip › PSAX/test/3105s1_55.png]

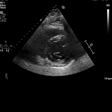

Supplement: Supplemental Information 2 [file peerj-cs-11-3161-s002.zip › PSAX/test/2564s1_69.png]

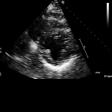

Supplement: Supplemental Information 2 [file peerj-cs-11-3161-s002.zip › PSAX/test/3190s1_30.png]

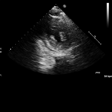

Supplement: Supplemental Information 2 [file peerj-cs-11-3161-s002.zip › PSAX/test/3521s1_49.png]

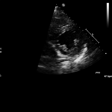

Supplement: Supplemental Information 2 [file peerj-cs-11-3161-s002.zip › PSAX/test/3413s1_43.png]

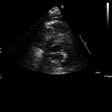

Supplement: Supplemental Information 2 [file peerj-cs-11-3161-s002.zip › PSAX/test/2257s1_40.png]

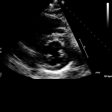

Supplement: Supplemental Information 2 [file peerj-cs-11-3161-s002.zip › PSAX/test/3190s1_31.png]

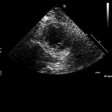

Supplement: Supplemental Information 2 [file peerj-cs-11-3161-s002.zip › PSAX/test/3105s1_54.png]

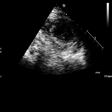

Supplement: Supplemental Information 2 [file peerj-cs-11-3161-s002.zip › PSAX/test/3325s1_65.png]

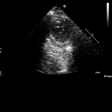

Supplement: Supplemental Information 2 [file peerj-cs-11-3161-s002.zip › PSAX/test/2515s1_20.png]

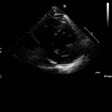

Supplement: Supplemental Information 2 [file peerj-cs-11-3161-s002.zip › PSAX/test/3339s2_25.png]

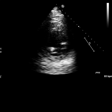

Supplement: Supplemental Information 2 [file peerj-cs-11-3161-s002.zip › PSAX/test/3503s1_48.png]

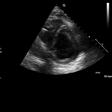

Supplement: Supplemental Information 2 [file peerj-cs-11-3161-s002.zip › PSAX/test/2670s1_37.png]

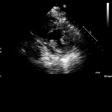

Supplement: Supplemental Information 2 [file peerj-cs-11-3161-s002.zip › PSAX/test/3325s1_64.png]

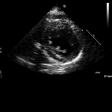

Supplement: Supplemental Information 2 [file peerj-cs-11-3161-s002.zip › PSAX/test/3105s1_51.png]

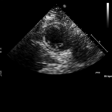

Supplement: Supplemental Information 2 [file peerj-cs-11-3161-s002.zip › PSAX/test/3105s1_53.png]

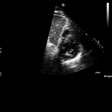

Supplement: Supplemental Information 2 [file peerj-cs-11-3161-s002.zip › PSAX/test/2515s1_37.png]

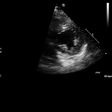

Supplement: Supplemental Information 2 [file peerj-cs-11-3161-s002.zip › PSAX/test/3413s1_44.png]

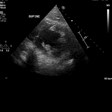

Supplement: Supplemental Information 2 [file peerj-cs-11-3161-s002.zip › PSAX/test/3181s1_41.png]

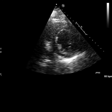

Supplement: Supplemental Information 2 [file peerj-cs-11-3161-s002.zip › PSAX/test/3305s1_19.png]

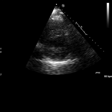

Supplement: Supplemental Information 2 [file peerj-cs-11-3161-s002.zip › PSAX/test/3305s1_18.png]

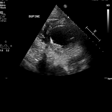

Supplement: Supplemental Information 2 [file peerj-cs-11-3161-s002.zip › PSAX/test/3181s1_40.png]

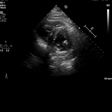

Supplement: Supplemental Information 2 [file peerj-cs-11-3161-s002.zip › PSAX/test/2200s1_38.png]

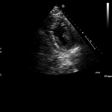

Supplement: Supplemental Information 2 [file peerj-cs-11-3161-s002.zip › PSAX/test/3216s2_29.png]

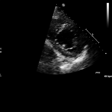

Supplement: Supplemental Information 2 [file peerj-cs-11-3161-s002.zip › PSAX/test/3413s1_45.png]

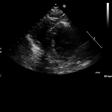

Supplement: Supplemental Information 2 [file peerj-cs-11-3161-s002.zip › PSAX/test/2965s1_29.png]

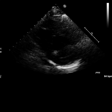

Supplement: Supplemental Information 2 [file peerj-cs-11-3161-s002.zip › PSAX/test/3339s2_27.png]

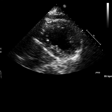

Supplement: Supplemental Information 2 [file peerj-cs-11-3161-s002.zip › PSAX/test/3105s1_52.png]

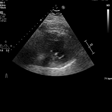

Supplement: Supplemental Information 2 [file peerj-cs-11-3161-s002.zip › PSAX/test/2564s1_63.png]

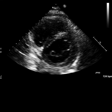

Supplement: Supplemental Information 2 [file peerj-cs-11-3161-s002.zip › PSAX/test/3659s1_51.png]

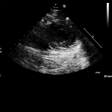

Supplement: Supplemental Information 2 [file peerj-cs-11-3161-s002.zip › PSAX/test/2965s1_30.png]

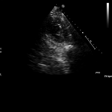

Supplement: Supplemental Information 2 [file peerj-cs-11-3161-s002.zip › PSAX/test/3216s2_30.png]

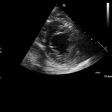

Supplement: Supplemental Information 2 [file peerj-cs-11-3161-s002.zip › PSAX/test/2670s1_38.png]

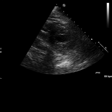

Supplement: Supplemental Information 2 [file peerj-cs-11-3161-s002.zip › PSAX/test/2670s1_39.png]

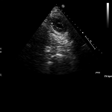

Supplement: Supplemental Information 2 [file peerj-cs-11-3161-s002.zip › PSAX/test/3216s2_31.png]

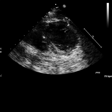

Supplement: Supplemental Information 2 [file peerj-cs-11-3161-s002.zip › PSAX/test/2965s1_31.png]

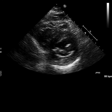

Supplement: Supplemental Information 2 [file peerj-cs-11-3161-s002.zip › PSAX/test/3600s1_27.png]

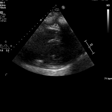

Supplement: Supplemental Information 2 [file peerj-cs-11-3161-s002.zip › PSAX/test/2564s1_60.png]

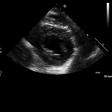

Supplement: Supplemental Information 2 [file peerj-cs-11-3161-s002.zip › PSAX/test/3600s1_25.png]

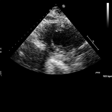

Supplement: Supplemental Information 2 [file peerj-cs-11-3161-s002.zip › PSAX/test/2948s2_53.png]

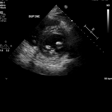

Supplement: Supplemental Information 2 [file peerj-cs-11-3161-s002.zip › PSAX/test/3181s1_73.png]

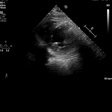

Supplement: Supplemental Information 2 [file peerj-cs-11-3161-s002.zip › PSAX/test/2200s1_37.png]

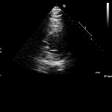

Supplement: Supplemental Information 2 [file peerj-cs-11-3161-s002.zip › PSAX/test/3521s1_69.png]

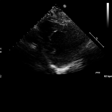

Supplement: Supplemental Information 2 [file peerj-cs-11-3161-s002.zip › PSAX/test/3339s2_28.png]

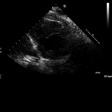

Supplement: Supplemental Information 2 [file peerj-cs-11-3161-s002.zip › PSAX/test/3659s1_53.png]

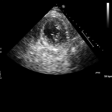

Supplement: Supplemental Information 2 [file peerj-cs-11-3161-s002.zip › PSAX/test/3132s1_24.png]

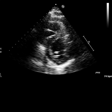

Supplement: Supplemental Information 2 [file peerj-cs-11-3161-s002.zip › PSAX/test/2281s1_17.png]

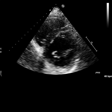

Supplement: Supplemental Information 2 [file peerj-cs-11-3161-s002.zip › PSAX/test/2948s2_56.png]

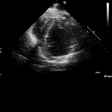

Supplement: Supplemental Information 2 [file peerj-cs-11-3161-s002.zip › PSAX/test/3375s1_63.png]

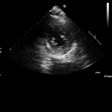

Supplement: Supplemental Information 2 [file peerj-cs-11-3161-s002.zip › PSAX/test/3521s1_50.png]

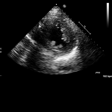

Supplement: Supplemental Information 2 [file peerj-cs-11-3161-s002.zip › PSAX/test/5966s1_40.png]

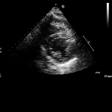

Supplement: Supplemental Information 2 [file peerj-cs-11-3161-s002.zip › PSAX/test/2281s1_16.png]

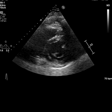

Supplement: Supplemental Information 2 [file peerj-cs-11-3161-s002.zip › PSAX/test/2564s1_70.png]

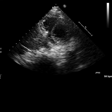

Supplement: Supplemental Information 2 [file peerj-cs-11-3161-s002.zip › PSAX/test/3659s1_54.png]

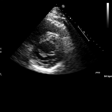

Supplement: Supplemental Information 2 [file peerj-cs-11-3161-s002.zip › PSAX/test/2987s1_44.png]

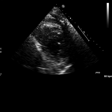

Supplement: Supplemental Information 2 [file peerj-cs-11-3161-s002.zip › PSAX/test/2987s1_37.png]

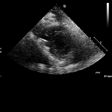

Supplement: Supplemental Information 2 [file peerj-cs-11-3161-s002.zip › PSAX/test/2415s1_34.png]

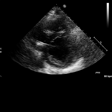

Supplement: Supplemental Information 2 [file peerj-cs-11-3161-s002.zip › PSAX/test/2415s1_35.png]

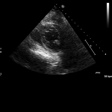

Supplement: Supplemental Information 2 [file peerj-cs-11-3161-s002.zip › PSAX/test/3230s1_19.png]

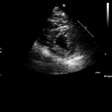

Supplement: Supplemental Information 2 [file peerj-cs-11-3161-s002.zip › PSAX/test/3670s1_34.png]

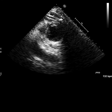

Supplement: Supplemental Information 2 [file peerj-cs-11-3161-s002.zip › PSAX/test/2894s1_54.png]

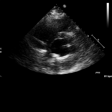

Supplement: Supplemental Information 2 [file peerj-cs-11-3161-s002.zip › PSAX/test/2415s1_36.png]

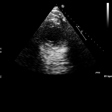

Supplement: Supplemental Information 2 [file peerj-cs-11-3161-s002.zip › PSAX/test/3503s1_26.png]

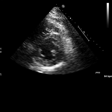

Supplement: Supplemental Information 2 [file peerj-cs-11-3161-s002.zip › PSAX/test/2987s1_35.png]

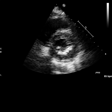

Supplement: Supplemental Information 2 [file peerj-cs-11-3161-s002.zip › PSAX/test/3670s1_27.png]

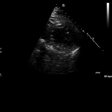

Supplement: Supplemental Information 2 [file peerj-cs-11-3161-s002.zip › PSAX/test/3492s3_30.png]

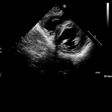

Supplement: Supplemental Information 2 [file peerj-cs-11-3161-s002.zip › PSAX/test/2398s1_17.png]

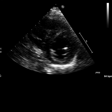

Supplement: Supplemental Information 2 [file peerj-cs-11-3161-s002.zip › PSAX/test/3283s1_61.png]

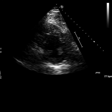

Supplement: Supplemental Information 2 [file peerj-cs-11-3161-s002.zip › PSAX/test/2241s1_28.png]

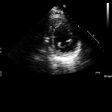

Supplement: Supplemental Information 2 [file peerj-cs-11-3161-s002.zip › PSAX/test/3332s1_34.png]

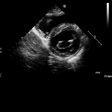

Supplement: Supplemental Information 2 [file peerj-cs-11-3161-s002.zip › PSAX/test/2398s1_16.png]

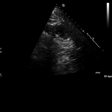

Supplement: Supplemental Information 2 [file peerj-cs-11-3161-s002.zip › PSAX/test/3492s3_31.png]

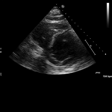

Supplement: Supplemental Information 2 [file peerj-cs-11-3161-s002.zip › PSAX/test/3230s1_23.png]

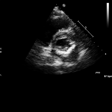

Supplement: Supplemental Information 2 [file peerj-cs-11-3161-s002.zip › PSAX/test/3670s1_32.png]

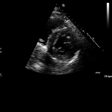

Supplement: Supplemental Information 2 [file peerj-cs-11-3161-s002.zip › PSAX/test/3492s3_27.png]

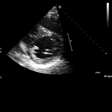

Supplement: Supplemental Information 2 [file peerj-cs-11-3161-s002.zip › PSAX/test/3153s1_47.png]

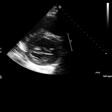

Supplement: Supplemental Information 2 [file peerj-cs-11-3161-s002.zip › PSAX/test/3153s1_46.png]

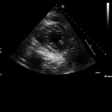

Supplement: Supplemental Information 2 [file peerj-cs-11-3161-s002.zip › PSAX/test/3230s1_20.png]

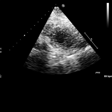

Supplement: Supplemental Information 2 [file peerj-cs-11-3161-s002.zip › PSAX/test/2405s1_53.png]

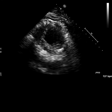

Supplement: Supplemental Information 2 [file peerj-cs-11-3161-s002.zip › PSAX/test/2894s1_53.png]
