# Supplementary material for: Quantification of left ventricular mass in multiple views of echocardiograms using model-agnostic meta learning in a few-shot setting
Source: PeerJ Comput Sci. 2025 Sep 16;11:e3161. doi: 10.7717/peerj-cs.3161 (PMC12453733; doi:10.7717/peerj-cs.3161)
Supplement: Supplemental Information 3 [file peerj-cs-11-3161-s003.docx]

**Supplementary Materials**

The proposed method can be used to calculate the left ventricular mass (LVM) shown in A to F of Fig. A1(Kristensen et al. 2022). In particular, the formula for F, which measures LVM by calculating three lines from four points in PLAX view, can be computed. In the formula, the terms represent $IVS$ as ventricular septal thickness at end-diastole, $LVID$ as LV internal diameter at end-diastole, and $LVPW$ as inferolateral (posterior) LV wall thickness at end-diastole(Lang et al. 2015). Additionally, using other views besides PLAX, such as C and E, the proposed method can be used to calculate the major values in the formula, and an additional process is needed to calculate LVM.


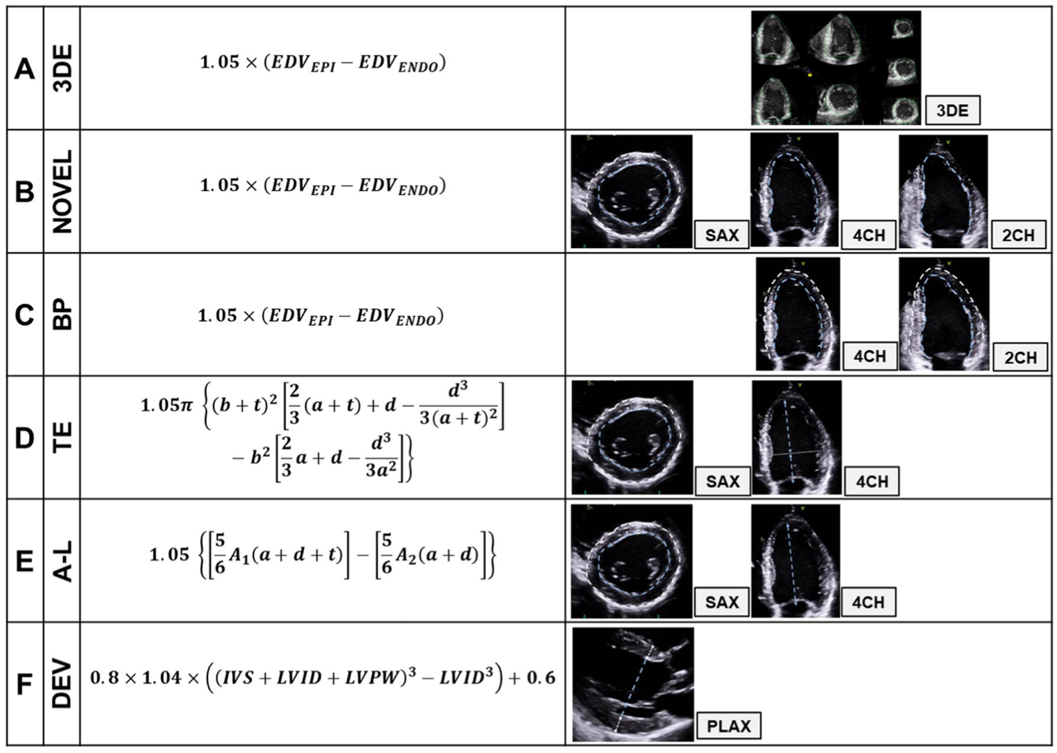


Fig. A1 Methods for measuring left ventricular mass in multiple views of echocardiogram(Kristensen et al. 2022). Reproduced under the terms of the Creative Commons Attribution 4.0 International License (CC BY 4.0; https://creativecommons.org/licenses/by/4.0/).

| **Dataset**  (view) | **Image** | **Image size**  (pixels) |
| --- | --- | --- |
| CAMUS(Leclerc et al. 2019b)  (A2C) | 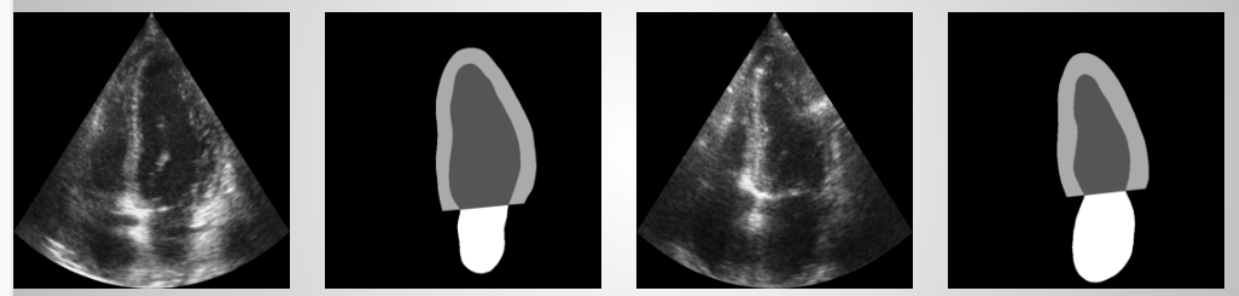 | 748x1,232 |
| CAMUS(Leclerc et al. 2019b)  (A4C) | 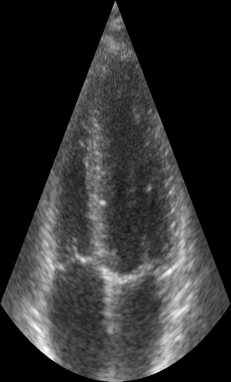 | 748x1,232 |
| EchoNet LVH(Duffy et al. 2022)  (PLAX) | 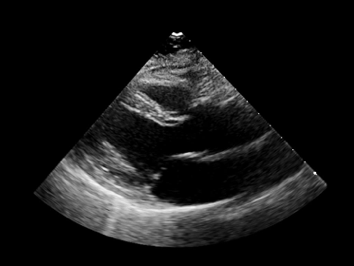 | Variable |
| TMED-2(Huang et al. 2022)  (PSAX) | 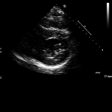 | 112x112 |

Fig. A2 Composition of the training set: A2C and A4C views from CAMUS(Leclerc et al. 2019a), PLAX view from EchoNet LVH(Duffy et al. 2022), and PSAX view from TMED-2(Huang et al. 2022) were used for the echocardiogram dataset. The proposed model was trained using k-shots sampling (e.g. k=5, 10, 20, and 30) from the total number of images during model-agnostic meta learning.


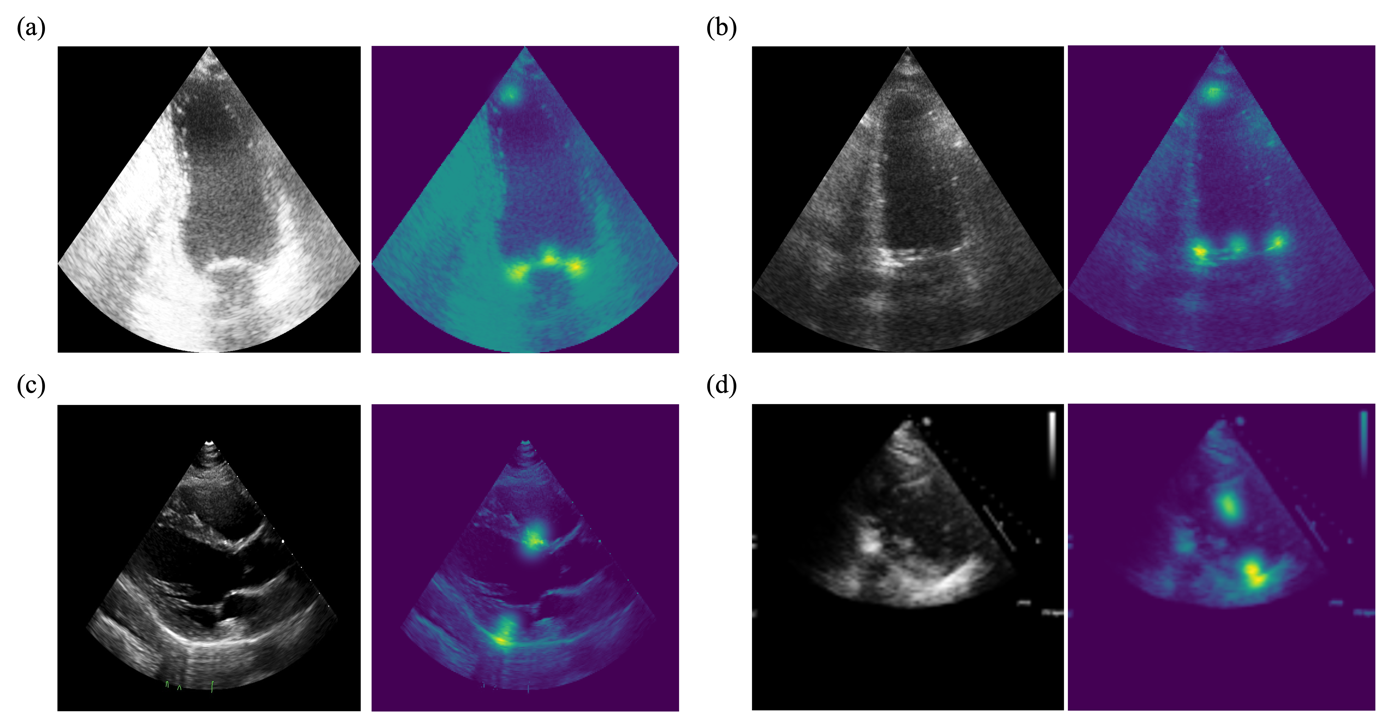


Fig. A3 Examples of the training set used for the proposed segmentation model in echocardiography: The proposed model utilized a heatmap-based point estimation method, generating labels (masks) with a standard deviation of 7 for gaussian distribution. On the left is the original image, and on the right is the image where the gaussian distribution is represented as a heatmap. (a) A2C view (b) A4C view (c) PLAX view (d) PSAX view


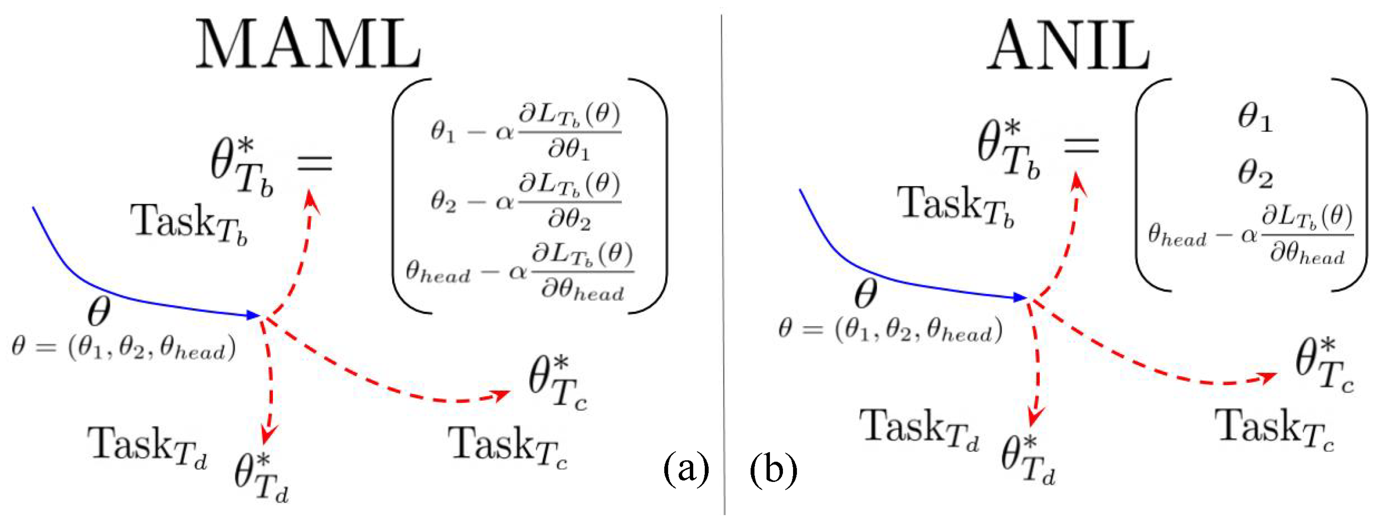


Fig. A4 An illustration of the differences between the MAML and ANIL methods(Tammisetti et al. 2024)*.* Reproduced under the terms of the Creative Commons Attribution 4.0 International License (CC BY 4.0; https://creativecommons.org/licenses/by/4.0/).

**References**

Duffy G, Cheng PP, Yuan N, He B, Kwan AC, Shun-Shin MJ, Alexander KM, Ebinger J, Lungren MP, and Rader FJJc. 2022. High-throughput precision phenotyping of left ventricular hypertrophy with cardiovascular deep learning. 7:386–395.

Finn C, Abbeel P, and Levine S. 2017. Model-agnostic meta-learning for fast adaptation of deep networks. International conference on machine learning: PMLR. p 1126–1135.

Huang Z, Long G, Wessler B, and Hughes MC. 2022. TMED 2: a dataset for semi-supervised classification of echocardiograms. DataPerf: Benchmarking Data for Data-Centric AI Workshop.

Kristensen CB, Myhr KA, Grund FF, Vejlstrup N, Hassager C, Mattu R, and Mogelvang R. 2022. A new method to quantify left ventricular mass by 2D echocardiography. *Scientific Reports* 12:9980.

Lang RM, Badano LP, Mor-Avi V, Afilalo J, Armstrong A, Ernande L, Flachskampf FA, Foster E, Goldstein SA, and Kuznetsova T. 2015. Recommendations for cardiac chamber quantification by echocardiography in adults: an update from the American Society of Echocardiography and the European Association of Cardiovascular Imaging. *European Heart Journal-Cardiovascular Imaging* 16:233–271.

Leclerc S, Smistad E, Pedrosa J, Østvik A, Cervenansky F, Espinosa F, Espeland T, Berg EAR, Jodoin P-M, and Grenier T. 2019a. Deep learning for segmentation using an open large-scale dataset in 2D echocardiography. *IEEE transactions on medical imaging* 38:2198–2210.

Leclerc S, Smistad E, Pedrosa J, Østvik A, Cervenansky F, Espinosa F, Espeland T, Berg EAR, Jodoin P-M, and Grenier TJItomi. 2019b. Deep learning for segmentation using an open large-scale dataset in 2D echocardiography. 38:2198–2210.

Li Z, Zhou F, Chen F, and Li HJapa. 2017. Meta-sgd: Learning to learn quickly for few-shot learning.

Park E, and Oliva JBJAinips. 2019. Meta-curvature. 32.

Tammisetti V, Bierzynski K, Stettinger G, Morales-Santos DP, Cuellar MP, and Molina-Solana M. 2024. LaANIL: ANIL with Look-Ahead Meta-Optimization and Data Parallelism. *Electronics* 13:1585.

Tammisetti V, Bierzynski K, Stettinger G, Morales-Santos DP, Cuellar MP, and Molina-Solana M. 2024. LaANIL: ANIL with Look-Ahead Meta-Optimization and Data Parallelism. *Electronics* 13:1585.
