# Supplementary material for: Quantification of left ventricular mass in multiple views of echocardiograms using model-agnostic meta learning in a few-shot setting
Source: PeerJ Comput Sci. 2025 Sep 16;11:e3161. doi: 10.7717/peerj-cs.3161 (PMC12453733; doi:10.7717/peerj-cs.3161)
Supplement: Supplemental Information 4 [file peerj-cs-11-3161-s004.docx]

Table A1 Qualitative results for the A2C view in echocardiograms using different model-agnostic meta learning methods.

| k-shot | Training method | Metric | A2C (CAMUS(Leclerc et al. 2019b)) | | | | |
| --- | --- | --- | --- | --- | --- | --- | --- |
|  |  |  | 1^st^ point | 2^nd^ point | 3^rd^ point | 4^th^ point | Avg. |
| 100 | Baseline | MDE | 7.96 ± 4.15 | 3.78 ± 3.81 | 3.15 ± 2.68 | 3.36 ± 2.31 | **4.56 ± 3.84** |
| 5 | FOMAML  (Finn et al. 2017) | MDE | 15.18 ± 6.47 | 11.68 ± 12.96 | 15.42 ± 18.40 | 12.20 ± 16.09 | 13.62 ± 14.36 |
|  | Meta-SGD  (Li et al. 2017) | MDE | 9.80 ± 5.10 | 9.51 ± 9.60 | 13.11 ± 17.36 | 15.93 ± 21.21 | 12.09 ± 14.82 |
|  | Meta-Curvature  (Park & Oliva 2019) | MDE | 13.57 ± 7.17 | 9.17 ± 16.46 | 5.95 ± 10.71 | 13.83 ± 21.20 | 10.62 ± 15.08 |
|  | ANIL  (Raghu et al., 2019) | MDE | 94.44 ± 28.53 | 19.57 ± 19.10 | 18.54 ± 20.64 | 24.77 ± 21.69 | 39.33 ± 22.49 |
| 10 | FOMAML  (Finn et al. 2017) | MDE | 14.93 ± 7.07 | 6.07 ± 6.07 | 5.76 ± 5.43 | 7.88 ± 10.41 | 8.66 ± 8.39 |
|  | Meta-SGD  (Li et al. 2017) | MDE | 18.30 ± 12.10 | 7.20 ± 11.18 | 8.59 ± 11.16 | 7.33 ± 10.49 | 10.35 ± 12.05 |
|  | Meta-Curvature  (Park & Oliva 2019) | MDE | 13.62 ± 7.33 | 7.06 ± 9.48 | 15.10 ± 24.27 | 9.73 ± 14.41 | 11.37 ± 15.49 |
|  | ANIL  (Raghu et al., 2019) | MDE | 87.44 ± 32.62 | 22.24 ± 24.91 | 17.65 ± 20.78 | 21.43 ± 23.05 | 37.19 ± 25.34 |
| 20 | FOMAML  (Finn et al. 2017) | MDE | 10.60 ± 4.04 | 4.50 ± 3.30 | 5.81 ± 4.24 | 6.44 ± 3.47 | 6.84 ± 4.38 |
|  | Meta-SGD  (Li et al. 2017) | MDE | 9.38 ± 3.96 | 7.10 ± 8.20 | 9.62 ± 11.43 | 7.88 ± 5.56 | 8.49 ± 7.80 |
|  | Meta-Curvature  (Park & Oliva 2019) | MDE | 15.16 ± 5.86 | 6.48 ± 10.15 | 6.60 ± 12.15 | 9.14 ± 7.88 | 9.34 ± 6.87 |
|  | ANIL  (Raghu et al., 2019) | MDE | 71.80 ± 43.43 | 30.15 ± 32.24 | 19.28 ± 19.31 | 18.55 ± 19.24 | 34.95 ± 28.56 |
| 30 | FOMAML  (Finn et al. 2017) | MDE | 9.67 ± 4.92 | 3.37 ± 2.40 | 3.05 ± 1.73 | 5.83 ± 3.61 | 5.48 ± 4.28 |
|  | Meta-SGD  (Li et al. 2017) | MDE | 9.97 ± 4.74 | 3.85 ± 3.27 | 3.05 ± 2.05 | 6.60 ± 4.96 | 5.87 ± 4.75 |
|  | Meta-Curvature  (Park & Oliva 2019) | MDE | 9.73 ± 4.44 | 4.71 ± 2.15 | 4.66 ± 2.83 | 6.57 ± 3.58 | 6.42 ± 3.92 |
|  | ANIL  (Raghu et al., 2019) | MDE | 19.59 ± 21.86 | 32.65 ± 36.00 | 29.23 ± 36.85 | 37.32 ± 43.20 | 29.70 ± 34.48 |

A2C, Apical 2-chamber; Avg, Average; MDE, Mean Distance Error; MAE, Mean Angle Error

**REFERENCES**

Duffy G, Cheng PP, Yuan N, He B, Kwan AC, Shun-Shin MJ, Alexander KM, Ebinger J, Lungren MP, and Rader FJJc. 2022. High-throughput precision phenotyping of left ventricular hypertrophy with cardiovascular deep learning. 7:386-395.

Finn C, Abbeel P, and Levine S. 2017. Model-agnostic meta-learning for fast adaptation of deep networks. International conference on machine learning: PMLR. p 1126-1135.

Huang Z, Long G, Wessler B, and Hughes MC. 2022. TMED 2: a dataset for semi-supervised classification of echocardiograms. DataPerf: Benchmarking Data for Data-Centric AI Workshop.

Kristensen CB, Myhr KA, Grund FF, Vejlstrup N, Hassager C, Mattu R, and Mogelvang R. 2022. A new method to quantify left ventricular mass by 2D echocardiography. *Scientific Reports* 12:9980.

Lang RM, Badano LP, Mor-Avi V, Afilalo J, Armstrong A, Ernande L, Flachskampf FA, Foster E, Goldstein SA, and Kuznetsova T. 2015. Recommendations for cardiac chamber quantification by echocardiography in adults: an update from the American Society of Echocardiography and the European Association of Cardiovascular Imaging. *European Heart Journal-Cardiovascular Imaging* 16:233-271.

Leclerc S, Smistad E, Pedrosa J, Østvik A, Cervenansky F, Espinosa F, Espeland T, Berg EAR, Jodoin P-M, and Grenier T. 2019a. Deep learning for segmentation using an open large-scale dataset in 2D echocardiography. *IEEE transactions on medical imaging* 38:2198-2210.

Leclerc S, Smistad E, Pedrosa J, Østvik A, Cervenansky F, Espinosa F, Espeland T, Berg EAR, Jodoin P-M, and Grenier TJItomi. 2019b. Deep learning for segmentation using an open large-scale dataset in 2D echocardiography. 38:2198-2210.

Li Z, Zhou F, Chen F, and Li HJapa. 2017. Meta-sgd: Learning to learn quickly for few-shot learning.

Park E, and Oliva JBJAinips. 2019. Meta-curvature. 32.

Raghu A, Raghu M, Bengio S, and Vinyals O. 2019. Rapid learning or feature reuse? towards understanding the effectiveness of maml. *arXiv preprint arXiv:190909157*.
