# Supplementary material for: Quantification of left ventricular mass in multiple views of echocardiograms using model-agnostic meta learning in a few-shot setting
Source: PeerJ Comput Sci. 2025 Sep 16;11:e3161. doi: 10.7717/peerj-cs.3161 (PMC12453733; doi:10.7717/peerj-cs.3161)
Supplement: Supplemental Information 5 [file peerj-cs-11-3161-s005.docx]

Table A2 Qualitative results for the A4C view in echocardiograms using different model-agnostic meta learning methods.

| k-shot | Training method | Metric | A4C (CAMUS(Leclerc et al. 2019b)) | | | | |
| --- | --- | --- | --- | --- | --- | --- | --- |
|  |  |  | 1^st^ point | 2^nd^ point | 3^rd^ point | 4^th^ point | Avg. |
| 100 | Baseline | MDE | 6.61 ± 4.21 | 4.29 ± 5.04 | 3.59 ± 3.27 | 6.19 ± 7.06 | **5.17 ± 5.23** |
| 5 | FOMAML  (Finn et al. 2017) | MDE | 9.24 ± 5.93 | 9.47 ± 8.72 | 10.79 ± 10.09 | 19.08 ± 13.55 | 12.14 ± 11.22 |
|  | Meta-SGD  (Li et al. 2017) | MDE | 11.74 ± 15.55 | 8.70 ± 6.58 | 13.22 ± 15.82 | 16.54 ± 13.48 | 12.55 ± 13.64 |
|  | Meta-Curvature  (Park & Oliva 2019) | MDE | 9.41 ± 6.01 | 8.40 ± 7.51 | 10.89 ± 10.82 | 14.69 ± 9.92 | 10.85 ± 10.73 |
|  | ANIL  (Raghu et al., 2019) | MDE | 41.43 ± 45.67 | 46.47 ± 42.03 | 48.19 ± 42.46 | 61.25 ± 49.93 | 49.33 ± 45.02 |
| 10 | FOMAML  (Finn et al. 2017) | MDE | 9.55 ± 5.79 | 6.51 ± 4.99 | 5.78 ± 5.64 | 11.70 ± 11.17 | 8.39 ± 7.68 |
|  | Meta-SGD  (Li et al. 2017) | MDE | 9.43 ± 5.43 | 5.08 ± 6.25 | 6.20 ± 7.14 | 10.53 ± 9.43 | 7.81 ± 7.53 |
|  | Meta-Curvature  (Park & Oliva 2019) | MDE | 9.40 ± 5.67 | 6.95 ± 5.39 | 8.66 ± 10.58 | 12.11 ± 10.59 | 9.29 ± 8.61 |
|  | ANIL  (Raghu et al., 2019) | MDE | 34.21 ± 40.18 | 39.32 ± 32.36 | 30.98 ± 26.45 | 44.54 ± 40.18 | 37.26 ± 34.79 |
| 20 | FOMAML  (Finn et al. 2017) | MDE | 9.01 ± 5.30 | 4.50 ± 3.34 | 5.13 ± 2.96 | 6.31 ± 3.23 | 6.24 ± 4.18 |
|  | Meta-SGD  (Li et al. 2017) | MDE | 8.74 ± 5.66 | 5.24 ± 7.37 | 5.85 ± 6.67 | 8.14 ± 8.42 | 6.99 ± 7.23 |
|  | Meta-Curvature  (Park & Oliva 2019) | MDE | 8.26 ± 5.54 | 6.40 ± 5.85 | 6.47 ± 8.56 | 8.61 ± 8.07 | 7.43 ± 7.17 |
|  | ANIL  (Raghu et al., 2019) | MDE | 9.02 ± 6.10 | 26.53 ± 26.90 | 19.95 ± 25.46 | 28.40 ± 22.61 | 20.98 ± 20.27 |
| 30 | FOMAML  (Finn et al. 2017) | MDE | 8.23 ± 9.31 | 4.05 ± 4.26 | 3.74 ± 3.94 | 5.78 ± 5.20 | 5.45 ± 6.30 |
|  | Meta-SGD  (Li et al. 2017) | MDE | 8.97 ± 7.09 | 4.25 ± 5.76 | 3.69 ± 3.79 | 5.89 ± 4.78 | 5.69 ± 5.84 |
|  | Meta-Curvature  (Park & Oliva 2019) | MDE | 8.54 ± 11.04 | 4.49 ± 3.14 | 4.57 ± 8.05 | 6.55 ± 5.86 | 6.04 ± 7.50 |
|  | ANIL  (Raghu et al., 2019) | MDE | 14.11 ± 41.51 | 23.13 ± 17.99 | 15.85 ± 18.94 | 10.69 ± 35.73 | 15.95 ± 28.54 |

A4C, Apical 4-chamber; Avg, Average; MDE, Mean Distance Error; MAE, Mean Angle Error

**REFERENCES**

Duffy G, Cheng PP, Yuan N, He B, Kwan AC, Shun-Shin MJ, Alexander KM, Ebinger J, Lungren MP, and Rader FJJc. 2022. High-throughput precision phenotyping of left ventricular hypertrophy with cardiovascular deep learning. 7:386-395.

Finn C, Abbeel P, and Levine S. 2017. Model-agnostic meta-learning for fast adaptation of deep networks. International conference on machine learning: PMLR. p 1126-1135.

Huang Z, Long G, Wessler B, and Hughes MC. 2022. TMED 2: a dataset for semi-supervised classification of echocardiograms. DataPerf: Benchmarking Data for Data-Centric AI Workshop.

Kristensen CB, Myhr KA, Grund FF, Vejlstrup N, Hassager C, Mattu R, and Mogelvang R. 2022. A new method to quantify left ventricular mass by 2D echocardiography. *Scientific Reports* 12:9980.

Lang RM, Badano LP, Mor-Avi V, Afilalo J, Armstrong A, Ernande L, Flachskampf FA, Foster E, Goldstein SA, and Kuznetsova T. 2015. Recommendations for cardiac chamber quantification by echocardiography in adults: an update from the American Society of Echocardiography and the European Association of Cardiovascular Imaging. *European Heart Journal-Cardiovascular Imaging* 16:233-271.

Leclerc S, Smistad E, Pedrosa J, Østvik A, Cervenansky F, Espinosa F, Espeland T, Berg EAR, Jodoin P-M, and Grenier T. 2019a. Deep learning for segmentation using an open large-scale dataset in 2D echocardiography. *IEEE transactions on medical imaging* 38:2198-2210.

Leclerc S, Smistad E, Pedrosa J, Østvik A, Cervenansky F, Espinosa F, Espeland T, Berg EAR, Jodoin P-M, and Grenier TJItomi. 2019b. Deep learning for segmentation using an open large-scale dataset in 2D echocardiography. 38:2198-2210.

Li Z, Zhou F, Chen F, and Li HJapa. 2017. Meta-sgd: Learning to learn quickly for few-shot learning.

Park E, and Oliva JBJAinips. 2019. Meta-curvature. 32.

Raghu A, Raghu M, Bengio S, and Vinyals O. 2019. Rapid learning or feature reuse? towards understanding the effectiveness of maml. *arXiv preprint arXiv:190909157*.
