# Supplementary material for: Quantification of left ventricular mass in multiple views of echocardiograms using model-agnostic meta learning in a few-shot setting
Source: PeerJ Comput Sci. 2025 Sep 16;11:e3161. doi: 10.7717/peerj-cs.3161 (PMC12453733; doi:10.7717/peerj-cs.3161)
Supplement: Supplemental Information 6 [file peerj-cs-11-3161-s006.docx]

Table A3 Qualitative results for the PLAX view in echocardiograms evaluated using the mean angle error (MAE) with different model-agnostic meta learning methods.

| k-shot | Training method | Metric | PLAX (EchoNet-LVH(Duffy et al. 2022)) | | | |
| --- | --- | --- | --- | --- | --- | --- |
|  |  |  | IVS | LVID | LVPW | Avg. |
| 100 | Baseline | MAE | 10.74 ± 9.42 | 5.11 ± 4.17 | 12.58 ± 11.73 | 9.48 ± 9.53 |
| 5 | FOMAML  (Finn et al. 2017) | MAE | 24.29 ± 21.17 | 7.79 ± 6.80 | 17.47 ± 16.84 | 16.52 ± 17.42 |
|  | Meta-SGD  (Li et al. 2017) | MAE | 28.75 ± 35.60 | 6.51 ± 5.10 | 23.97 ± 24.71 | 19.74 ± 26.87 |
|  | Meta-Curvature  (Park & Oliva 2019) | MAE | 22.41 ± 20.82 | 6.06 ± 5.72 | 23.71 ± 17.67 | 17.39 ± 17.96 |
|  | ANIL  (Raghu et al., 2019) | MAE | 50.05 ± 44.21 | 18.58 ± 28.56 | 30.05 ± 30.97 | 32.89 ± 37.47 |
| 10 | FOMAML  (Finn et al. 2017) | MAE | 16.63 ± 20.99 | 11.17 ± 18.63 | 27.90 ± 22.62 | 18.57 ± 21.88 |
|  | Meta-SGD  (Li et al. 2017) | MAE | 17.00 ± 19.61 | 6.53 ± 4.72 | 14.25 ± 13.50 | 12.59 ± 14.65 |
|  | Meta-Curvature  (Park & Oliva 2019) | MAE | 14.89 ± 19.07 | 5.67 ± 6.57 | 10.87 ± 11.51 | 10.48 ± 13.89 |
|  | ANIL  (Raghu et al., 2019) | MAE | 55.88 ± 49.90 | 14.95 ± 16.88 | 40.76 ± 38.93 | 37.20 ± 41.32 |
| 20 | FOMAML  (Finn et al. 2017) | MAE | 13.11 ± 9.76 | 5.32 ± 4.17 | 13.69 ± 10.28 | 10.71 ± 9.32 |
|  | Meta-SGD  (Li et al. 2017) | MAE | 10.88 ± 10.25 | 5.29 ± 4.51 | 9.18 ± 6.43 | 8.45 ± 7.79 |
|  | Meta-Curvature  (Park & Oliva 2019) | MAE | 11.39 ± 13.53 | 8.54 ± 21.11 | 10.27 ± 8.05 | 10.07 ± 15.20 |
|  | ANIL  (Raghu et al., 2019) | MAE | 36.85 ± 43.33 | 21.18 ± 35.98 | 30.57 ± 33.01 | 29.53 ± 38.12 |
| 30 | FOMAML  (Finn et al. 2017) | MAE | 8.43 ± 6.58 | 4.26 ± 3.40 | 10.33 ± 7.68 | **7.67 ± 6.64** |
|  | Meta-SGD  (Li et al. 2017) | MAE | 7.62 ± 6.45 | 5.46 ± 4.58 | 12.62 ± 18.50 | 8.57 ± 11.96 |
|  | Meta-Curvature  (Park & Oliva 2019) | MAE | 11.85 ± 16.2463 | 4.44 ± 3.37 | 8.18 ± 6.47 | 8.16 ± 10.68 |
|  | ANIL  (Raghu et al., 2019) | MAE | 37.02 ± 29.59 | 16.05 ± 2163 | 29.34 ± 25.72 | 27.47 ± 21.19 |

PLAX, Parasternal Long Axes; Avg, Average; MAE, Mean Angle Error; IVS, Intraventricular Septum; LVID, Left Ventricular Internal Dimension LVPW, Left Ventricular Posterior Wall

**REFERENCES**

Duffy G, Cheng PP, Yuan N, He B, Kwan AC, Shun-Shin MJ, Alexander KM, Ebinger J, Lungren MP, and Rader FJJc. 2022. High-throughput precision phenotyping of left ventricular hypertrophy with cardiovascular deep learning. 7:386-395.

Finn C, Abbeel P, and Levine S. 2017. Model-agnostic meta-learning for fast adaptation of deep networks. International conference on machine learning: PMLR. p 1126-1135.

Huang Z, Long G, Wessler B, and Hughes MC. 2022. TMED 2: a dataset for semi-supervised classification of echocardiograms. DataPerf: Benchmarking Data for Data-Centric AI Workshop.

Kristensen CB, Myhr KA, Grund FF, Vejlstrup N, Hassager C, Mattu R, and Mogelvang R. 2022. A new method to quantify left ventricular mass by 2D echocardiography. *Scientific Reports* 12:9980.

Lang RM, Badano LP, Mor-Avi V, Afilalo J, Armstrong A, Ernande L, Flachskampf FA, Foster E, Goldstein SA, and Kuznetsova T. 2015. Recommendations for cardiac chamber quantification by echocardiography in adults: an update from the American Society of Echocardiography and the European Association of Cardiovascular Imaging. *European Heart Journal-Cardiovascular Imaging* 16:233-271.

Leclerc S, Smistad E, Pedrosa J, Østvik A, Cervenansky F, Espinosa F, Espeland T, Berg EAR, Jodoin P-M, and Grenier T. 2019a. Deep learning for segmentation using an open large-scale dataset in 2D echocardiography. *IEEE transactions on medical imaging* 38:2198-2210.

Leclerc S, Smistad E, Pedrosa J, Østvik A, Cervenansky F, Espinosa F, Espeland T, Berg EAR, Jodoin P-M, and Grenier TJItomi. 2019b. Deep learning for segmentation using an open large-scale dataset in 2D echocardiography. 38:2198-2210.

Li Z, Zhou F, Chen F, and Li HJapa. 2017. Meta-sgd: Learning to learn quickly for few-shot learning.

Park E, and Oliva JBJAinips. 2019. Meta-curvature. 32.

Raghu A, Raghu M, Bengio S, and Vinyals O. 2019. Rapid learning or feature reuse? towards understanding the effectiveness of maml. *arXiv preprint arXiv:190909157*.
