# Supplementary material for: Quantification of left ventricular mass in multiple views of echocardiograms using model-agnostic meta learning in a few-shot setting
Source: PeerJ Comput Sci. 2025 Sep 16;11:e3161. doi: 10.7717/peerj-cs.3161 (PMC12453733; doi:10.7717/peerj-cs.3161)
Supplement: Supplemental Information 7 [file peerj-cs-11-3161-s007.docx]

Table A4 Qualitative results for the PLAX view in echocardiograms evaluated using the mean distance error (MDE) with different model-agnostic meta learning methods.

| k-shot | Training method | Metric | PLAX (EchoNet-LVH(Duffy et al. 2022)) | | | | |
| --- | --- | --- | --- | --- | --- | --- | --- |
|  |  |  | 1^st^ point | 2^nd^ point | 3^rd^ point | 4^th^ point | Avg. |
| 100 | Baseline | MDE | 4.19 ± 4.05 | 4.51 ± 4.03 | 5.27 ± 3.58 | 6.48 ± 3.81 | 5.11 ± 3.96 |
| 5 | FOMAML  (Finn et al. 2017) | MDE | 7.74 ± 11.66 | 6.98 ± 9.14 | 7.45 ± 4.84 | 7.38 ± 4.83 | 7.39 ± 8.13 |
|  | Meta-SGD  (Li et al. 2017) | MDE | 5.64 ± 4.28 | 6.67 ± 3.79 | 7.83 ± 5.23 | 7.30 ± 5.06 | 6.86 ± 4.68 |
|  | Meta-Curvature  (Park & Oliva 2019) | MDE | 5.31 ± 4.60 | 6.42 ± 6.04 | 8.58 ± 4.93 | 7.63 ± 5.44 | 6.99 ± 5.40 |
|  | ANIL  (Raghu et al., 2019) | MDE | 18.01 ± 17.19 | 16.75 ± 13.77 | 15.23 ± 14.60 | 12.43 ± 8.21 | 15.60 ± 13.44 |
| 10 | FOMAML  (Finn et al. 2017) | MDE | 4.71 ± 3.39 | 4.73 ± 2.67 | 10.71 ± 9.53 | 10.34 ± 9.40 | 7.62 ± 7.58 |
|  | Meta-SGD  (Li et al. 2017) | MDE | 6.32 ± 5.77 | 5.91 ± 5.33 | 7.38 ± 4.84 | 7.41 ± 4.61 | 6.76 ± 5.18 |
|  | Meta-Curvature  (Park & Oliva 2019) | MDE | 5.12 ± 4.08 | 5.31 ± 5.68 | 6.73 ± 3.92 | 7.01 ± 4.56 | 6.04 ± 4.67 |
|  | ANIL  (Raghu et al., 2019) | MDE | 16.97 ± 17.08 | 14.09 ± 12.32 | 12.53 ± 11.29 | 12.30 ± 10.07 | 13.97 ± 12.69 |
| 20 | FOMAML  (Finn et al. 2017) | MDE | 4.18 ± 2.60 | 4.49 ± 3.10 | 6.15 ± 3.66 | 6.93 ± 4.39 | 5.44 ± 3.67 |
|  | Meta-SGD  (Li et al. 2017) | MDE | 5.18 ± 4.38 | 5.05 ± 3.88 | 6.24 ± 4.29 | 6.76 ± 4.59 | 5.81 ± 4.34 |
|  | Meta-Curvature  (Park & Oliva 2019) | MDE | 4.78 ± 5.99 | 5.24 ± 7.70 | 6.17 ± 3.84 | 7.06 ± 4.46 | 5.81 ± 5.74 |
|  | ANIL  (Raghu et al., 2019) | MDE | 14.63 ± 13.48 | 16.97 ± 20.00 | 13.55 ± 12.65 | 13.10 ± 11.91 | 14.56 ± 14.51 |
| 30 | FOMAML  (Finn et al. 2017) | MDE | 4.08 ± 2.56 | 4.04 ± 2.53 | 5.65 ± 3.19 | 6.45 ± 3.85 | 5.06 ± 3.24 |
|  | Meta-SGD  (Li et al. 2017) | MDE | 4.65 ± 2.32 | 4.37 ± 2.69 | 6.29 ± 4.67 | 6.46 ± 4.08 | 5.45 ± 3.68 |
|  | Meta-Curvature  (Park & Oliva 2019) | MDE | 4.83 ± 4.96 | 5.31 ± 4.44 | 5.72 ± 3.49 | 6.38 ± 4.02 | 5.56 ± 4.28 |
|  | ANIL  (Raghu et al., 2019) | MDE | 10.93 ± 10.74 | 10.62 ± 9.00 | 12.67 ± 13.99 | 10.30 ± 9.63 | 11.13 ± 10.84 |

PLAX, Parasternal Long Axes; Avg, Average; MDE, Mean Distance Error

**REFERENCES**

Duffy G, Cheng PP, Yuan N, He B, Kwan AC, Shun-Shin MJ, Alexander KM, Ebinger J, Lungren MP, and Rader FJJc. 2022. High-throughput precision phenotyping of left ventricular hypertrophy with cardiovascular deep learning. 7:386-395.

Finn C, Abbeel P, and Levine S. 2017. Model-agnostic meta-learning for fast adaptation of deep networks. International conference on machine learning: PMLR. p 1126-1135.

Huang Z, Long G, Wessler B, and Hughes MC. 2022. TMED 2: a dataset for semi-supervised classification of echocardiograms. DataPerf: Benchmarking Data for Data-Centric AI Workshop.

Kristensen CB, Myhr KA, Grund FF, Vejlstrup N, Hassager C, Mattu R, and Mogelvang R. 2022. A new method to quantify left ventricular mass by 2D echocardiography. *Scientific Reports* 12:9980.

Lang RM, Badano LP, Mor-Avi V, Afilalo J, Armstrong A, Ernande L, Flachskampf FA, Foster E, Goldstein SA, and Kuznetsova T. 2015. Recommendations for cardiac chamber quantification by echocardiography in adults: an update from the American Society of Echocardiography and the European Association of Cardiovascular Imaging. *European Heart Journal-Cardiovascular Imaging* 16:233-271.

Leclerc S, Smistad E, Pedrosa J, Østvik A, Cervenansky F, Espinosa F, Espeland T, Berg EAR, Jodoin P-M, and Grenier T. 2019a. Deep learning for segmentation using an open large-scale dataset in 2D echocardiography. *IEEE transactions on medical imaging* 38:2198-2210.

Leclerc S, Smistad E, Pedrosa J, Østvik A, Cervenansky F, Espinosa F, Espeland T, Berg EAR, Jodoin P-M, and Grenier TJItomi. 2019b. Deep learning for segmentation using an open large-scale dataset in 2D echocardiography. 38:2198-2210.

Li Z, Zhou F, Chen F, and Li HJapa. 2017. Meta-sgd: Learning to learn quickly for few-shot learning.

Park E, and Oliva JBJAinips. 2019. Meta-curvature. 32.

Raghu A, Raghu M, Bengio S, and Vinyals O. 2019. Rapid learning or feature reuse? towards understanding the effectiveness of maml. *arXiv preprint arXiv:190909157*.
