# Supplementary material for: Quantification of left ventricular mass in multiple views of echocardiograms using model-agnostic meta learning in a few-shot setting
Source: PeerJ Comput Sci. 2025 Sep 16;11:e3161. doi: 10.7717/peerj-cs.3161 (PMC12453733; doi:10.7717/peerj-cs.3161)
Supplement: Supplemental Information 8 [file peerj-cs-11-3161-s008.docx]

Table A5 Qualitative results for the PSAX view in echocardiograms evaluated using the mean angle error (MAE) with different model-agnostic meta learning methods.

| k-shot | Training method | Metric | PSAX (TMED-2(Huang et al. 2022)) | | | |
| --- | --- | --- | --- | --- | --- | --- |
|  |  |  | IVS | LVID | LVPW | Avg. |
| 100 | Baseline | MAE | 23.85 ± 18.69 | 12.98 ± 11.12 | 26.85 ± 33.37 | 26.85 ± 33.37 |
| 5 | FOMAML  (Finn et al. 2017) | MAE | 26.61 ± 19.38 | 18.96 ± 19.87 | 26.37 ± 25.92 | 23.98 ± 22.14 |
|  | Meta-SGD  (Li et al. 2017) | MAE | 23.12 ± 18.95 | 27.13 ± 28.95 | 40.17 ± 41.73 | 30.14 ± 32.03 |
|  | Meta-Curvature  (Park & Oliva 2019) | MAE | 21.62 ± 20.03 | 22.15 ± 23.61 | 27.39 ± 19.31 | 23.72 ± 21.16 |
|  | ANIL  (Raghu et al., 2019) | MAE | 28.75 ± 31.73 | 33.81 ± 37.05 | 38.88 ± 38.81 | 33.81 ± 36.11 |
| 10 | FOMAML  (Finn et al. 2017) | MAE | 30.69 ± 36.36 | 19.38 ± 26.79 | 35.43 ± 31.59 | 28.50 ± 32.42 |
|  | Meta-SGD  (Li et al. 2017) | MAE | 23.34 ± 20.12 | 17.61 ± 20.87 | 24.59 ± 20.12 | 21.85 ± 20.53 |
|  | Meta-Curvature  (Park & Oliva 2019) | MAE | 18.01 ± 15.39 | 15.45 ± 17.79 | 21.56 ± 15.73 | 18.34 ± 16.48 |
|  | ANIL  (Raghu et al., 2019) | MAE | 56.01 ± 60.48 | 28.80 ± 37.90 | 37.98 ± 32.05 | 40.93 ± 46.42 |
| 20 | FOMAML  (Finn et al. 2017) | MAE | 18.19 ± 13.80 | 13.20 ± 14.33 | 24.60 ± 25.30 | 18.66 ± 19.10 |
|  | Meta-SGD  (Li et al. 2017) | MAE | 17.47 ± 15.93 | 14.86 ± 14.41 | 27.13 ± 25.38 | 19.82 ± 19.85 |
|  | Meta-Curvature  (Park & Oliva 2019) | MAE | 17.45 ± 14.23 | 15.17 ± 12.04 | 22.41 ± 20.68 | 18.34 ± 16.30 |
|  | ANIL  (Raghu et al., 2019) | MAE | 46.95 ± 47.74 | 28.42 ± 36.17 | 38.35 ± 33.11 | 37.91 ± 40.10 |
| 30 | FOMAML  (Finn et al. 2017) | MAE | 16.70 ± 14.38 | 13.82 ± 9.71 | 15.16 ± 11.41 | 15.23 ± 12.01 |
|  | Meta-SGD  (Li et al. 2017) | MAE | 13.24 ± 10.36 | 12.44 ± 8.85 | 14.90 ± 11.01 | 13.53 ± 10.13 |
|  | Meta-Curvature  (Park & Oliva 2019) | MAE | 13.86 ± 10.75 | 12.21 ± 9.54 | 12.12 ± 10.76 | **12.73 ± 10.36** |
|  | ANIL  (Raghu et al., 2019) | MAE | 31.62 ± 29.59 | 23.03 ± 27.30 | 32.41 ± 27.20 | 29.02 ± 28.28 |

PSAX, Parasternal Short Axes; Avg, Average; MAE, Mean Angle Error; IVS, Intraventricular Septum; LVID, Left Ventricular Internal Dimension LVPW, Left Ventricular Posterior Wall

**REFERENCES**

Duffy G, Cheng PP, Yuan N, He B, Kwan AC, Shun-Shin MJ, Alexander KM, Ebinger J, Lungren MP, and Rader FJJc. 2022. High-throughput precision phenotyping of left ventricular hypertrophy with cardiovascular deep learning. 7:386-395.

Finn C, Abbeel P, and Levine S. 2017. Model-agnostic meta-learning for fast adaptation of deep networks. International conference on machine learning: PMLR. p 1126-1135.

Huang Z, Long G, Wessler B, and Hughes MC. 2022. TMED 2: a dataset for semi-supervised classification of echocardiograms. DataPerf: Benchmarking Data for Data-Centric AI Workshop.

Kristensen CB, Myhr KA, Grund FF, Vejlstrup N, Hassager C, Mattu R, and Mogelvang R. 2022. A new method to quantify left ventricular mass by 2D echocardiography. *Scientific Reports* 12:9980.

Lang RM, Badano LP, Mor-Avi V, Afilalo J, Armstrong A, Ernande L, Flachskampf FA, Foster E, Goldstein SA, and Kuznetsova T. 2015. Recommendations for cardiac chamber quantification by echocardiography in adults: an update from the American Society of Echocardiography and the European Association of Cardiovascular Imaging. *European Heart Journal-Cardiovascular Imaging* 16:233-271.

Leclerc S, Smistad E, Pedrosa J, Østvik A, Cervenansky F, Espinosa F, Espeland T, Berg EAR, Jodoin P-M, and Grenier T. 2019a. Deep learning for segmentation using an open large-scale dataset in 2D echocardiography. *IEEE transactions on medical imaging* 38:2198-2210.

Leclerc S, Smistad E, Pedrosa J, Østvik A, Cervenansky F, Espinosa F, Espeland T, Berg EAR, Jodoin P-M, and Grenier TJItomi. 2019b. Deep learning for segmentation using an open large-scale dataset in 2D echocardiography. 38:2198-2210.

Li Z, Zhou F, Chen F, and Li HJapa. 2017. Meta-sgd: Learning to learn quickly for few-shot learning.

Park E, and Oliva JBJAinips. 2019. Meta-curvature. 32.

Raghu A, Raghu M, Bengio S, and Vinyals O. 2019. Rapid learning or feature reuse? towards understanding the effectiveness of maml. *arXiv preprint arXiv:190909157*.
