# Supplementary material for: Quantification of left ventricular mass in multiple views of echocardiograms using model-agnostic meta learning in a few-shot setting
Source: PeerJ Comput Sci. 2025 Sep 16;11:e3161. doi: 10.7717/peerj-cs.3161 (PMC12453733; doi:10.7717/peerj-cs.3161)
Supplement: Supplemental Information 9 [file peerj-cs-11-3161-s009.docx]

**Table A6** Qualitative results for the PSAX view in echocardiograms evaluated using the mean distance error (MDE) with different model-agnostic meta learning methods.

| k-shot | Training method | Metric | PSAX (TMED-2(Huang et al. 2022)) | | | | |
| --- | --- | --- | --- | --- | --- | --- | --- |
|  |  |  | 1^st^ point | 2^nd^ point | 3^rd^ point | 4^th^ point | Avg. |
| 100 | Baseline | MDE | 9.32 ± 6.06 | 6.33 ± 5.34 | 6.28 ± 4.98 | 7.59 ± 5.79 | 7.38 ± 5.67 |
| 5 | FOMAML  (Finn et al. 2017) | MDE | 13.61 ± 7.78 | 10.31 ± 7.72 | 10.57 ± 8.10 | 9.30 ± 6.67 | 10.95 ± 7.73 |
|  | Meta-SGD  (Li et al. 2017) | MDE | 12.81 ± 7.88 | 10.93 ± 7.48 | 15.41 ± 16.99 | 12.07 ± 10.86 | 12.80 ± 11.53 |
|  | Meta-Curvature  (Park & Oliva 2019) | MDE | 12.35 ± 7.70 | 9.68 ± 6.81 | 9.79 ± 8.03 | 9.28 ± 5.60 | 10.27 ± 7.17 |
|  | ANIL  (Raghu et al., 2019) | MDE | 18.59 ± 10.71 | 15.56 ± 9.44 | 22.74 ± 13.52 | 20.25 ± 16.02 | 19.28 ± 12.42 |
| 10 | FOMAML  (Finn et al. 2017) | MDE | 14.40 ± 12.64 | 10.59 ± 10.80 | 9.91 ± 12.79 | 9.36 ± 7.16 | 11.06 ± 11.21 |
|  | Meta-SGD(Li et al. 2017) | MDE | 9.64 ± 7.46 | 7.38 ± 5.60 | 9.54 ± 6.93 | 10.05 ± 7.90 | 9.15 ± 7.08 |
|  | Meta-Curvature  (Park & Oliva 2019) | MDE | 11.13 ± 7.85 | 9.16 ± 8.08 | 9.46 ± 8.04 | 9.74 ± 7.84 | 9.87 ± 7.96 |
|  | ANIL  (Raghu et al., 2019) | MDE | 21.59 ± 17.41 | 16.24 ± 13.60 | 13.83 ± 7.72 | 13.54 ± 7.70 | 16.27 ± 11.61 |
| 20 | FOMAML  (Finn et al. 2017) | MDE | 9.03 ± 5.37 | 7.81 ± 5.03 | 9.08 ± 11.18 | 10.12 ± 6.42 | 9.01 ± 7.44 |
|  | Meta-SGD  (Li et al. 2017) | MDE | 8.44 ± 5.06 | 7.20 ± 4.18 | 9.99 ± 10.80 | 9.81 ± 5.64 | 8.86 ± 6.99 |
|  | Meta-Curvature  (Park & Oliva 2019) | MDE | 8.91 ± 4.56 | 8.57 ± 5.25 | 9.55 ± 6.65 | 9.63 ± 6.23 | 9.16 ± 5.73 |
|  | ANIL  (Raghu et al., 2019) | MDE | 15.99 ± 11.46 | 12.21 ± 9.37 | 12.06 ± 9.67 | 12.55 ± 10.98 | 13.20 ± 10.37 |
| 30 | FOMAML  (Finn et al. 2017) | MDE | 7.76 ± 4.05 | 6.65 ± 4.09 | 8.46 ± 4.69 | 8.65 ± 4.74 | 7.88 ± 4.46 |
|  | Meta-SGD  (Li et al. 2017) | MDE | 7.11 ± 3.97 | 6.46 ± 3.68 | 7.09 ± 3.46 | 7.77 ± 3.99 | **7.11 ± 3.80** |
|  | Meta-Curvature  (Park & Oliva 2019) | MDE | 7.65 ± 4.37 | 7.10 ± 4.40 | 8.05 ± 4.62 | 8.33 ± 4.85 | 7.78 ± 4.57 |
|  | ANIL  (Raghu et al., 2019) | MDE | 12.22 ± 13.05 | 9.17 ± 12.12 | 12.63 ± 11.96 | 12.76 ± 12.00 | 11.70 ± 12.28 |

PSAX, Parasternal Short Axes; Avg, Average; MDE, Mean Distance Error

**REFERENCES**

Duffy G, Cheng PP, Yuan N, He B, Kwan AC, Shun-Shin MJ, Alexander KM, Ebinger J, Lungren MP, and Rader FJJc. 2022. High-throughput precision phenotyping of left ventricular hypertrophy with cardiovascular deep learning. 7:386-395.

Finn C, Abbeel P, and Levine S. 2017. Model-agnostic meta-learning for fast adaptation of deep networks. International conference on machine learning: PMLR. p 1126-1135.

Huang Z, Long G, Wessler B, and Hughes MC. 2022. TMED 2: a dataset for semi-supervised classification of echocardiograms. DataPerf: Benchmarking Data for Data-Centric AI Workshop.

Kristensen CB, Myhr KA, Grund FF, Vejlstrup N, Hassager C, Mattu R, and Mogelvang R. 2022. A new method to quantify left ventricular mass by 2D echocardiography. *Scientific Reports* 12:9980.

Lang RM, Badano LP, Mor-Avi V, Afilalo J, Armstrong A, Ernande L, Flachskampf FA, Foster E, Goldstein SA, and Kuznetsova T. 2015. Recommendations for cardiac chamber quantification by echocardiography in adults: an update from the American Society of Echocardiography and the European Association of Cardiovascular Imaging. *European Heart Journal-Cardiovascular Imaging* 16:233-271.

Leclerc S, Smistad E, Pedrosa J, Østvik A, Cervenansky F, Espinosa F, Espeland T, Berg EAR, Jodoin P-M, and Grenier T. 2019a. Deep learning for segmentation using an open large-scale dataset in 2D echocardiography. *IEEE transactions on medical imaging* 38:2198-2210.

Leclerc S, Smistad E, Pedrosa J, Østvik A, Cervenansky F, Espinosa F, Espeland T, Berg EAR, Jodoin P-M, and Grenier TJItomi. 2019b. Deep learning for segmentation using an open large-scale dataset in 2D echocardiography. 38:2198-2210.

Li Z, Zhou F, Chen F, and Li HJapa. 2017. Meta-sgd: Learning to learn quickly for few-shot learning.

Park E, and Oliva JBJAinips. 2019. Meta-curvature. 32.

Raghu A, Raghu M, Bengio S, and Vinyals O. 2019. Rapid learning or feature reuse? towards understanding the effectiveness of maml. *arXiv preprint arXiv:190909157*.
